# Neuroimaging Studies of Acupuncture on Low Back Pain: A Systematic Review

**DOI:** 10.3389/fnins.2021.730322

**Published:** 2021-09-20

**Authors:** Qiao Wen, Peihong Ma, Xiaohui Dong, Ruirui Sun, Lei Lan, Tao Yin, Yuzhu Qu, Yalan Liu, Qingqing Xiao, Fang Zeng

**Affiliations:** Acupuncture and Tuina School, Acupuncture and Brain Science Research Center, Chengdu University of Traditional Chinese Medicine, Chengdu, China

**Keywords:** acupuncture, neuroimaging, fMRI, low back pain, systematic review

## Abstract

**Objectives:** This study was conducted in order to investigate the study design and main outcomes of acupuncture neuroimaging studies on low back pain (LBP).

**Methods:** Neuroimaging studies of acupuncture on LBP were collected from three English databases such as PubMed and four Chinese databases such as China National Knowledge Infrastructure (CNKI) from inception to December 31, 2020. Study selection, data extraction, and assessment of risk of bias were performed independently by two investigators. The quality of studies was appraised with the Cochrane's risk of bias tools. Information on basic information, methodology, and brain imaging data were extracted.

**Results:** The literature search returned 310 potentially eligible studies and 19 articles met inclusion criteria; 78.9% of studies chose manual acupuncture as the intervention, 89.5% of studies evaluated functional changes elicited by acupuncture, and 68.4% of studies used resting-state fMRI as imaging condition. The most frequently reported acupuncture-induced brain alterations of LBP patients were in the prefrontal cortex, insula, cerebellum, primary somatosensory cortex, and anterior cingulate cortex. There was a significant correlation between improved clinical outcomes and changes in the brain.

**Conclusions:** The results suggested that improving abnormal structure and functional activities in the brain of the LBP patient is an important mechanism of acupuncture treatment for LBP. The brain regions involved in acupuncture analgesia for LBP were mainly located in the pain matrix, default mode network (DMN), salience network (SN), and descending pain modulatory system (DPMS). However, it was difficult to draw a generalized conclusion due to the heterogeneity of study designs. Further well-designed multimodal neuroimaging studies investigating the mechanism of acupuncture on LBP are warranted.

## Introduction

Low back pain (LBP) is a common disorder defined as the pain between the lower rib margins and the buttock creases (Kreiner et al., 2020). The lifetime and mean point prevalence of LBP were around 40 and 20%, respectively (Hoy et al., 2012; Calvo-Muñoz et al., 2013). LBP is the leading cause of disability that accounts for around 60.1 million years lived with disability (YLD) globally, and affects people of all ages (GBD 2016 Disease Injury Incidence Prevalence Collaborators, 2017). As an effective and safe therapy, acupuncture has been widely used for LBP. Many systematic reviews indicate that acupuncture is effective in relieving pain and improving function in LBP patients (Xu et al., 2013; Chou et al., 2017; Fuentes et al., 2020; Su et al., 2021). Since the first acupuncture neuroimaging study for LBP published in 2007 (Ji et al., 2007), there has been a surge of interest in investigating the therapeutic mechanism that underpins acupuncture treatment for LBP with neuroimaging technologies.

In the past 14 years, around 20 acupuncture neuroimaging studies for LBP have been published. These studies provide visualized and real-time evidence for understanding the mechanism of acupuncture treatment for LBP. However, the methodology issues of these studies greatly differed from each other. For example, some studies selected manual acupuncture (MA) as acupuncture modality, while others used electroacupuncture (EA). Some studies were performed with resting-state fMRI (rs-fMRI), while others with the task-state fMRI (ts-fMRI). Some focused on the functional changes elicited by acupuncture, while others centered on the structural alterations resulting from acupuncture. Therefore, this review aimed to (1) analyze the methodology issues of the published acupuncture neuroimaging studies on LBP, (2) summarize the core brain regions involved in acupuncture analgesia for LBP, and (3) provide references for future studies and the application of the neuroimaging results in the clinic.

## Methods

### Literature Search

The following seven databases were searched from inception to December 31, 2020: PubMed, EMBASE, Cochrane database, China National Knowledge Infrastructure (CNKI), Chinese Biomedical Literature (CBM), Chongqing VIP Database (VIP, Chinese Database), and Wanfang Database (WF, Chinese Database). The language was restricted to English or Chinese. The keywords were a combination of “low back pain,” “acupuncture,” and “neuroimaging technologies.” The search strategy for each of the electronic databases queried is shown in Supplementary Table 1. Additional studies were found by screening references of included articles and relevant reviews.

### Eligibility Criteria

Studies were eligible for inclusion if they (1) were peer-reviewed original research conducted in LBP patients, (2) applied acupuncture as the intervention, and (3) used structural and functional magnetic resonance imaging (sMRI, fMRI), positron emission tomography (PET), electroencephalography (EEG), and functional near-infrared spectroscopy (fNIRS), etc.

We excluded the experimental pain model, conference abstracts, case reports, protocols, animal study, monograph, and reviews.

### Study Selection, Data Extraction, and Assessment of Risk of Bias

Study selection, data extraction, and assessment of risk of bias were performed independently by two investigators (XD and YQ). Two Cochrane's risk of bias tools (Sterne et al., 2016, 2019) were utilized for evaluating the risk of bias of included randomized controlled trials (RCTs) and non-RCTs, respectively. Discrepancies were addressed by discussion or consulting a third party (PM).

For the eligible studies, we retrieved the following: (1) basic information: first author's name, year of publication, country, and diagnosis; (2) methodology: characteristics and sample size of participants, study design, acupuncture intervention (treatment courses, acupoints, manipulation modality, and needle sensation), imaging modality, and analysis methods; and (3) brain imaging data.

### Synthesis of Results

There is a great difference in the methodology issues of the included studies, and a meta-analysis was considered inappropriate. A descriptive analysis was presented to summarize (1) the brain alterations of LBP patients, (2) acupuncture-induced brain alterations, (3) brain functional alterations induced by MA and EA, and (4) acupuncture-related brain activities in resting-state and task-state fMRI.

## Results

### Study Selection and Description

**Figure 1:**
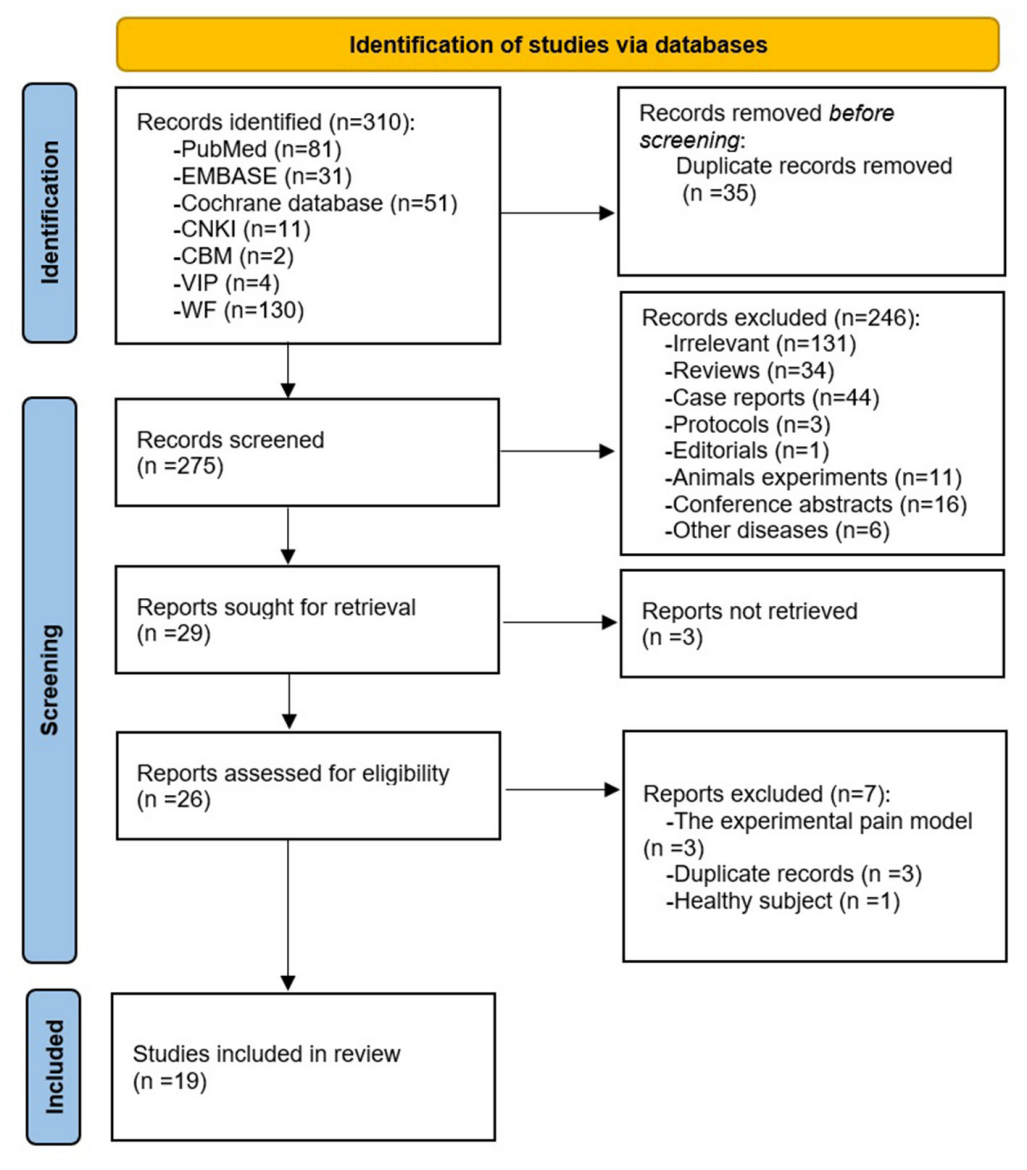
The flow diagram of literature search and screening process.

The Preferred Reporting Items for Systematic Reviews and Meta-Analyses (PRISMA) flow diagram (Page et al., 2021) of literature search and screening process is shown in Figure 1. A total of 310 results were yielded from the databases. After deduplication and screening phases, 19 studies (Ji et al., 2007; Junhai et al., 2008; Yongsong et al., 2011; Li et al., 2012, 2014; Guoqiang et al., 2014; Jialiang et al., 2014; Tao et al., 2015; Lin and Xianmo, 2017; Yijun et al., 2017; Makary et al., 2018; Lee et al., 2019; Tu et al., 2019; Xiang et al., 2019; Yan et al., 2019; Kim et al., 2020; Lan et al., 2020; Liu et al., 2020; Yu et al., 2020) between 2007 and 2020 were incorporated in this review (Table 1). Among the articles, 14 studies were performed in China (Ji et al., 2007; Junhai et al., 2008; Yongsong et al., 2011; Li et al., 2012, 2014; Guoqiang et al., 2014; Jialiang et al., 2014; Tao et al., 2015; Lin and Xianmo, 2017; Yijun et al., 2017; Xiang et al., 2019; Yan et al., 2019; Lan et al., 2020; Liu et al., 2020), three were conducted in the USA (Tu et al., 2019; Kim et al., 2020; Yu et al., 2020), and two were conducted in South Korea (Makary et al., 2018; Lee et al., 2019). Five kinds of diseases were involved in these studies: chronic low back pain (Li et al., 2014; Tu et al., 2019; Xiang et al., 2019; Yan et al., 2019; Kim et al., 2020; Yu et al., 2020), non-specific low back pain (Makary et al., 2018; Lee et al., 2019), lumbar disc herniation (Yongsong et al., 2011; Guoqiang et al., 2014; Jialiang et al., 2014; Tao et al., 2015; Lin and Xianmo, 2017; Yijun et al., 2017; Lan et al., 2020), sciatica (Ji et al., 2007; Li et al., 2012; Liu et al., 2020), and low back and leg pain (Junhai et al., 2008).

**Table 1:** Characteristics of the included studies.

**No**.	**Author (year) country**	**Diseases**	**Design**	***N* (intervention/control)**	**Age**	**Treatment courses**	**Intervention/control (acupoints)**	**Manipulation modality**	**Needle sensation**	**Imaging modality**	**Analytical approaches**
1	Ji et al. (2007) China	Sciatica	Non-RCT	12	32–45	30 min	(A) Affected leg (B) Healthy leg (GB34, GB39)	EA	Not Depicted	ts-fMRI	Brain activation
2	Junhai et al. (2008) China	Low back and leg pain	Non-RCT	12	32–45	30 min	(A) Palm temperature increased B) Palm temperature decreased (GB34, GB39)	EA	Deqi	ts-fMRI	Brain activation
3	Yongsong et al. (2011) China	Lumbar disc herniation	Non-RCT	20 (10/10)	35–69	Not depicted	(A) Patients (B) HC (the lumbago acupoint)	MA	Deqi	rs-fMRI	Voxel-wise FC
4	Li et al. (2012) China	Chronic sciatica	Non-RCT	20 (10/10)	32–45	22 d	(A) Patients (acupoint selection by syndrome differentiation) B) HC	EA	Deqi	rs-fMRI	ICA
5	Li et al. (2014) China	Chronic low back pain	Non-RCT	30 (20/10)	LBP 38.1 ± 6.4 HC 37.7 ± 5.1	30 min	(A) Patients (BL23, ashi point, GV3, BL40 and KI3) (B) HC	MA	Deqi	rs-fMRI	ICA, correlation with VAS
6	Jialiang et al. (2014) China	Lumbar disc herniation	RCT	15 (7/8)	35–75	25 min	(A) Balanced acupuncture (the lumbago acupoint) (B) Body acupuncture (BL25, BL26, ashi point)	MA	Not depicted	rs-fMRI	ReHo
7	Guoqiang et al. (2014) China	Lumbar disc herniation	Non-RCT	12	55–70	3 min 45 s	(A) Acupuncture (GB41)	MA	Not depicted	ts-fMRI	Brain activation
8	Tao et al. (2015) China	Lumbar disc herniation	RCT	30 (14/16)	30–70	20 min	(A) Balanced acupuncture (the lumbago acupoint) (B) Body acupuncture (BL25, BL26, ashi point)	MA	Not depicted	rs-fMRI	ReHo
9	Yijun et al. (2017) China	L5 nerve root pain	Non-RCT	10	20–65	3 min	(A) Acupuncture (gentong 2)	MA	Deqi	rs-fMRI and ts-fMRI	Brain activation
10	Lin and Xianmo (2017) China	Lumbar disc herniation	RCT	92 (42/50)	25–60	15 min	(A) Yaosanzhen acupuncture (BL23, BL25, BL40) (B) Body acupuncture (BL25, BL26, ashi point)	MA	Not depicted	rs-fMRI	ReHo
11	Makary et al. (2018) South Korea	Non-specific low back pain	RCT	56 (33/23)	38.4 ± 12.7	7 min	(A) Verum (B) Phantom acupuncture (ST36, SP11, SP13)	MA	Deqi	ts-fMRI	Brain activation, correlation with ANS response, belief in acupuncture effectiveness, VAS, and MASS Index
12	Tu et al. (2019) USA	Chronic low back pain	RCT	50 (24/26)	Verum 39.0 ± 12.6 Sham 40.0 ± 13.7	4 w	(A) Verum (GV3, BL23, BL40, KI3, ashi points) (B) Sham (non-acupoints treated by a Streitberger placebo acupuncture needle)	MA	Deqi	rs-fMRI	ROI-wise FC, MVPA
13	Xiang et al. (2019) China	Chronic low back pain	Non-RCT	12	18–65	8 min	(A) Verum (ankle zone 5) (B) Sham (tactile stimulation)	MA	Not deqi	rs-fMRI	ALFF/fALFF, correlation with VAS
14	Lee et al. (2019)South Korea	Non-specific low back pain	RCT	56 (33/23)	38.4 ± 12.7	7 min	(A) Verum (B) Phantom acupuncture (ST36, SP11, SP13)	MA	Deqi	rs-fMRI	ICA, correlation with VAS
15	Yan et al. (2019) China	Chronic low back pain	Non-RCT	57 (16/16/25)	LBP 46.4 ± 10.0 HC 40.0 ± 9.8	4 w	(A) Kidney deficiency patients (B) Non-kidney deficiency patients (acupoint selection by syndrome differentiation) C) HC	EA	Not depicted	rs-fMRI	ReHo
16	Yu et al. (2020) USA	Chronic low back pain	RCT	54 (14/13/14/13)	18–60	15 min	(A) “Augmented context” (interaction with the acupuncturist) verum (B) “Limited context” (converse with patients as little as possible) verum (GV3, BL23, BL40, KI3, ashi points) (C) “Augmented context” sham D) “Limited context” sham (non-acupoints treated by a Streitberger placebo acupuncture needle)	MA	Deqi	rs-fMRI	ROI-wise FC, correlation with VAS
17	Liu et al. (2020) China	Chronic sciatica	Non-RCT	27 (12/15)	35–85	4 w	(A) Patients (acupoint selection by syndrome differentiation) (B) HC	MA	Deqi	rs-fMRI	ReHo/Voxel-wise FC, correlation between VAS, SBI, and RDQS
18	Kim et al. (2020) USA	Chronic low back pain	RCT	128 (18/18/19/23/50)	18–60	4 w	(A) Verum (GV3, BL23, BL40, KI3, ashi points) (B) Sham (using a Streitberger placebo acupuncture needle) (C) Mock laser acupuncture (D) usual care E) HC	MA	Not depicted	VBM, DTI	GMV, FA
19	Lan et al. (2020) China	L5 nerve root pain	RCT	40 (20/20)	18–60	1 w	(A) Acupuncture 1 (gentong 2) (B) Lumbar traction + acupuncture 2 (acupoint selection by syndrome differentiation)	MA	Deqi	DTI	AD, FA

*AD, axial diffusivity; ANS, autonomic nervous system; (f)ALFF, (functional) amplitude of low-frequency fluctuations; d, day; DTI, diffusion tensor imaging; EA, electroacupuncture; FA, fractional anisotropy; FC, functional connectivity; HC, healthy controls; ICA, independent component analysis; LBP, low back pain; MA, manual acupuncture; MASS, Massachusetts general hospital acupuncture sensation scale; min, minute; MVPA, multivariate pattern analysis; n, number; RCT: randomized controlled trial; ReHo, regional homogeneity; rs-fMRI, resting-state fMRI; s, second; SBI, sciatica bothersomeness index; sham, sham acupuncture; ts-fMRI, task-state fMRI; VAS, visual analog scale; VBM, voxel-based morphometry; verum, verum acupuncture; w, week*.

### Details of Methodology

#### Participants

A total of 613 LBP patients and 120 healthy controls (HC) were investigated. Six studies (Yongsong et al., 2011; Li et al., 2012, 2014; Yan et al., 2019; Kim et al., 2020; Liu et al., 2020) compared LBP patients and HC, while the remaining 13 studies (Ji et al., 2007; Junhai et al., 2008; Guoqiang et al., 2014; Jialiang et al., 2014; Tao et al., 2015; Lin and Xianmo, 2017; Yijun et al., 2017; Makary et al., 2018; Lee et al., 2019; Tu et al., 2019; Xiang et al., 2019; Lan et al., 2020; Yu et al., 2020) only recruited patients. Thirteen studies included LBP patients between 18 and 85 years of age, and the mean age of patients in the other six studies (in which the age range was not reported) ranged from 25.7 to 56.4 years. Eighteen studies described the gender of the patients (289 female and 280 male). One study (Lan et al., 2020) did not mention the gender of the patients. In the six studies that compared LBP patients and HC, the maximum and minimum sample sizes of LBP/HC were 23/50 and 10/10 per group, respectively. The average sample size of LBP/HC was 16/20 per group. In the 13 studies that only recruited patients, the maximum and minimum sample sizes were 50 and 7 per group, respectively. The average sample size of LBP was 20 per group.

#### Study Design

Nine RCTs and 10 non-RCTs were involved in these studies. The control groups in RCTs mainly included sham acupuncture (Makary et al., 2018; Lee et al., 2019; Tu et al., 2019; Kim et al., 2020; Lan et al., 2020; Yu et al., 2020), different acupoints (Jialiang et al., 2014; Tao et al., 2015; Lin and Xianmo, 2017), and usual care (Kim et al., 2020). Among the studies that compared verum acupuncture with sham acupuncture, the sham acupuncture methods included (1) phantom acupuncture (watched a recorded video clip of a verum stimulation to avoid any somatosensory afference) (Makary et al., 2018; Lee et al., 2019), (2) Streitberger placebo acupuncture needle at sham acupoints (Tu et al., 2019; Yu et al., 2020), (3) Streitberger placebo acupuncture needle at acupoints (Kim et al., 2020), and (4) mock laser acupuncture (Kim et al., 2020). Among the studies that compared different acupoints, LBP patients were randomly assigned into two different acupoints groups (balanced acupuncture/body acupuncture, yaosanzhen acupuncture/body acupuncture) (Jialiang et al., 2014; Tao et al., 2015; Lin and Xianmo, 2017).

#### Details of Interventions

Fifteen studies used MA, while four trials used EA. Thirteen studies focused on the instant effect of acupuncture, and the duration of stimulation was from 3 to 30 min (Ji et al., 2007; Junhai et al., 2008; Yongsong et al., 2011; Guoqiang et al., 2014; Jialiang et al., 2014; Li et al., 2014; Tao et al., 2015; Lin and Xianmo, 2017; Yijun et al., 2017; Makary et al., 2018; Lee et al., 2019; Xiang et al., 2019; Yu et al., 2020). Six studies focused on the cumulative effect of acupuncture, and the treatment courses were from 1 to 8 weeks (Li et al., 2012; Tu et al., 2019; Yan et al., 2019; Kim et al., 2020; Lan et al., 2020; Liu et al., 2020). Four studies chose single acupoint (Yongsong et al., 2011; Guoqiang et al., 2014; Yijun et al., 2017; Xiang et al., 2019), two studies used two acupoints (Ji et al., 2007; Junhai et al., 2008), nine studies applied a combination of three or more acupoints (Jialiang et al., 2014; Li et al., 2014; Tao et al., 2015; Lin and Xianmo, 2017; Makary et al., 2018; Lee et al., 2019; Tu et al., 2019; Kim et al., 2020; Yu et al., 2020), and four chose acupoints by syndrome differentiation (Li et al., 2012; Yan et al., 2019; Lan et al., 2020; Liu et al., 2020). The most frequently used acupoints were ashi points, BL23, BL40, and BL25 (Supplementary Figure 1). Eleven studies emphasized the needle sensation (Deqi) during acupuncture stimulation (Junhai et al., 2008; Yongsong et al., 2011; Li et al., 2012, 2014; Yijun et al., 2017; Makary et al., 2018; Lee et al., 2019; Tu et al., 2019; Lan et al., 2020; Liu et al., 2020; Yu et al., 2020).

#### Imaging Condition and Analysis

Magnetic resonance imaging (MRI) was applied in all studies to measure neuronal activity and brain structure in LBP patients treated by acupuncture.

Two studies evaluated structure changes. One study employed the single modality of diffusion tensor imaging (DTI) to investigate fractional anisotropy (FA) and axial diffusivity (AD) (Lan et al., 2020). One study combined DTI and voxel-based morphology to explore both gray matter volume (GMV) and FA (Kim et al., 2020).

Seventeen studies evaluated functional changes. One study combined resting and task-state fMRI (Yijun et al., 2017). Five studies used ts-fMRI (Ji et al., 2007; Junhai et al., 2008; Guoqiang et al., 2014; Yijun et al., 2017; Makary et al., 2018). Three studies were performed with a block design (Li et al., 2014; Sterne et al., 2016, 2019). Two studies were performed with event-related experimental paradigm (Guoqiang et al., 2014; Yijun et al., 2017). The needle is stimulated continuously for a duration from 45 s to 2 min during two to five blocks.

Thirteen studies (Yongsong et al., 2011; Li et al., 2012, 2014; Jialiang et al., 2014; Tao et al., 2015; Lin and Xianmo, 2017; Yijun et al., 2017; Lee et al., 2019; Tu et al., 2019; Xiang et al., 2019; Yan et al., 2019; Liu et al., 2020; Yu et al., 2020) used rs-fMRI to evaluate regional homogeneity (ReHo) (five studies) (Jialiang et al., 2014; Tao et al., 2015; Lin and Xianmo, 2017; Yan et al., 2019; Liu et al., 2020), functional connectivity (FC) (four studies) (Yongsong et al., 2011; Tu et al., 2019; Liu et al., 2020; Yu et al., 2020), independent component analysis (ICA) (three studies) (Li et al., 2012, 2014; Lee et al., 2019), and amplitude of low-frequency fluctuations/fractional amplitude of low-frequency fluctuations (ALFF/fALFF) (one study) (Xiang et al., 2019), one of which used machine learning approaches to predict acupuncture treatment responses (Tu et al., 2019).

#### Results of Risk-of-Bias Assessments

The results of risk-of-bias assessments are presented in Supplementary Tables 2, 3.

Of the nine RCTs, two studies were rated as “low” overall risks of bias (Tu et al., 2019; Yu et al., 2020), whereas seven studies were rated as “some concerns” of overall risks of bias due to concerns regarding the randomization process (Jialiang et al., 2014; Tao et al., 2015; Lin and Xianmo, 2017; Makary et al., 2018; Lee et al., 2019; Kim et al., 2020; Lan et al., 2020) and missing outcome data (Jialiang et al., 2014; Tao et al., 2015; Lin and Xianmo, 2017).

Of the 10 non-RCTs, nine studies were rated as “low” overall risks of bias (Tu et al., 2019; Yu et al., 2020). However, one study was rated as “no information” of overall risks of bias due to concerns regarding the missing outcome data (Guoqiang et al., 2014).

### Brain Imaging Data

Brain imaging data of frequently reported brain regions are summarized in Table 2.

**Table 2:** Brain imaging data of frequently reported brain regions.

**Brain regions**					
LBP vs. HC	PFC	ACC	Precuneus	Insula	SI
Acupuncture-induced brain alterations					
Function	PFC	Insula	Cerebellum	SI	ACC
Structure	SI				
MA vs. EA					
MA	PFC	Cerebellum	Insula	SI	ACC
EA	PFC	ACC	STG	SI	Insula
rs-fMRI vs. ts-fMRI					
rs-fMRI	PFC	Cerebellum	Insula	SI	ACC
ts-fMRI	Cerebellum	Insula	SI	SII	PFC

*ACC, anterior cingulate cortex; HC, healthy controls; LBP, low back pain; PFC, prefrontal cortex; rs-fMRI, resting-state fMRI; SI, primary somatosensory cortex; SII, secondary somatosensory cortex; STG, superior temporal gyrus; ts-fMRI, task-state fMRI*.

#### Brain Alterations of LBP Patients

Five studies reported the functional and structural alterations of LBP patients. Compared with HC, LBP patients mainly showed alterations in PFC (four studies) (Li et al., 2012, 2014; Yan et al., 2019; Liu et al., 2020), ACC (three studies) (Li et al., 2012, 2014; Liu et al., 2020), precuneus (three studies) (Li et al., 2012, 2014; Yan et al., 2019), insula (two studies) (Yan et al., 2019; Liu et al., 2020), and the primary somatosensory cortex (SI) (two studies) (Yan et al., 2019; Kim et al., 2020).

**Figure 2:**
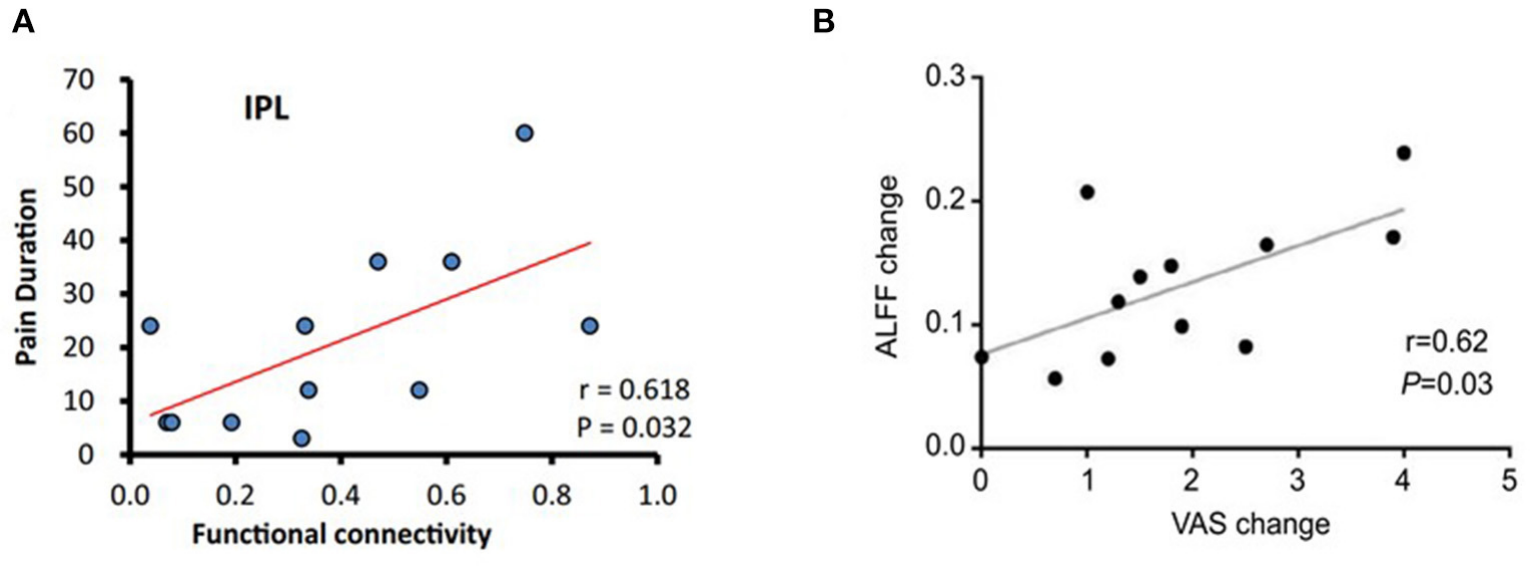
Correlation analysis in some studies (Xiang et al., 2019; Liu et al., 2020). **(A)** The PCC-seeded FC was positively correlated with pain duration in the right inferior parietal lobule at baseline in LBP patients. **(B)** There was a significant correlation between ALFF change in the left insular and VAS change after acupuncture treatment. ALFF, amplitude of low-frequency fluctuation; FC, functional connectivity; LBP, low back pain; PCC, posterior cingulate cortex; VAS, visual analog scale/score.

Correlation analyses showed a positive correlation between posterior cingulate cortex (PCC)–right inferior parietal lobule FC and the duration of sciatica (Liu et al., 2020) (Figure 2A).

#### Acupuncture-Related Brain Alterations in LBP Patients

The most frequently reported acupuncture-related brain functional alterations in LBP patients were in PFC (12 studies) (Junhai et al., 2008; Yongsong et al., 2011; Li et al., 2012, 2014; Guoqiang et al., 2014; Jialiang et al., 2014; Lin and Xianmo, 2017; Makary et al., 2018; Lee et al., 2019; Tu et al., 2019; Yan et al., 2019; Liu et al., 2020), insula (nine studies) (Ji et al., 2007; Junhai et al., 2008; Yongsong et al., 2011; Guoqiang et al., 2014; Makary et al., 2018; Lee et al., 2019; Tu et al., 2019; Xiang et al., 2019; Liu et al., 2020), cerebellum (nine studies) (Ji et al., 2007; Junhai et al., 2008; Yongsong et al., 2011; Guoqiang et al., 2014; Jialiang et al., 2014; Tao et al., 2015; Lin and Xianmo, 2017; Yijun et al., 2017; Liu et al., 2020), SI (eight studies) (Ji et al., 2007; Junhai et al., 2008; Yongsong et al., 2011; Jialiang et al., 2014; Tao et al., 2015; Lin and Xianmo, 2017; Makary et al., 2018; Yan et al., 2019), and ACC (seven studies) (Ji et al., 2007; Junhai et al., 2008; Yongsong et al., 2011; Li et al., 2012, 2014; Makary et al., 2018; Liu et al., 2020).

Two studies found corresponding results regarding regional structural changes in LBP patients after acupuncture treatment. Following a 4-week course of acupuncture, GVM was reduced and white matter FA was increased in the SI–back, and the changes were associated with improvements in tactile acuity over the back (Kim et al., 2020; Lan et al., 2020).

One study found that pretreatment FC between the medial prefrontal cortex (mPFC) and other brain regions can predict treatment responsiveness of acupuncture on LBP patients (Tu et al., 2019). In addition, baseline periaqueductal gray (PAG)–amygdala FC can predict bothersomeness reduction after acupuncture treatments (Yu et al., 2020). There was a significant correlation between mean ALFF change in the left insula, FC of the PFC–insula, and the decreased pain (Lee et al., 2019; Xiang et al., 2019) (Figure 2B).

#### MA- and EA-Related Brain Alterations in LBP Patients

Of the 15 studies that used MA, the most frequently reported EA-related brain alterations in LBP patients were in the PFC (nine studies) (Yongsong et al., 2011; Guoqiang et al., 2014; Jialiang et al., 2014; Li et al., 2014; Lin and Xianmo, 2017; Makary et al., 2018; Lee et al., 2019; Tu et al., 2019; Liu et al., 2020), cerebellum (seven studies) (Yongsong et al., 2011; Guoqiang et al., 2014; Jialiang et al., 2014; Tao et al., 2015; Lin and Xianmo, 2017; Yijun et al., 2017; Liu et al., 2020), insula (seven studies) (Yongsong et al., 2011; Guoqiang et al., 2014; Makary et al., 2018; Lee et al., 2019; Tu et al., 2019; Xiang et al., 2019; Liu et al., 2020), SI (five studies) (Yongsong et al., 2011; Jialiang et al., 2014; Tao et al., 2015; Lin and Xianmo, 2017; Makary et al., 2018), and ACC (four studies) (Yongsong et al., 2011; Li et al., 2014; Makary et al., 2018; Liu et al., 2020).

Of the four studies that used EA, the most frequently reported EA-related brain alterations in LBP patients were in the PFC (four studies) (Ji et al., 2007; Junhai et al., 2008; Li et al., 2012; Yan et al., 2019), ACC (three studies) (Ji et al., 2007; Junhai et al., 2008; Li et al., 2012), superior temporal gyrus (STG) (three studies) (Ji et al., 2007; Junhai et al., 2008; Yan et al., 2019), SI (three studies) (Ji et al., 2007; Junhai et al., 2008; Yan et al., 2019), and insula (two studies) (Ji et al., 2007; Junhai et al., 2008).

#### Acupuncture-Related Brain Activities in Resting-State and Task-State fMRI

Of the 13 studies that used resting-state fMRI, the most frequently reported acupuncture-related brain alterations of LBP patients were in the PFC (nine studies) (Yongsong et al., 2011; Li et al., 2012, 2014; Jialiang et al., 2014; Lin and Xianmo, 2017; Lee et al., 2019; Tu et al., 2019; Yan et al., 2019; Liu et al., 2020), cerebellum (five studies) (Yongsong et al., 2011; Jialiang et al., 2014; Tao et al., 2015; Lin and Xianmo, 2017; Liu et al., 2020), insula (five studies) (Yongsong et al., 2011; Lee et al., 2019; Tu et al., 2019; Xiang et al., 2019; Liu et al., 2020), SI (five studies) (Yongsong et al., 2011; Jialiang et al., 2014; Tao et al., 2015; Lin and Xianmo, 2017; Yan et al., 2019), and ACC (four studies) (Yongsong et al., 2011; Li et al., 2012, 2014; Liu et al., 2020).

Of the five studies that used task-state fMRI, the most frequently reported acupuncture-related brain alterations of LBP patients were in the cerebellum (four studies) (Ji et al., 2007; Junhai et al., 2008; Guoqiang et al., 2014; Yijun et al., 2017), insula (four studies) (Ji et al., 2007; Junhai et al., 2008; Guoqiang et al., 2014; Makary et al., 2018), SI (three studies) (Ji et al., 2007; Junhai et al., 2008; Makary et al., 2018), secondary somatosensory cortex (SII) (three studies) (Ji et al., 2007; Junhai et al., 2008; Makary et al., 2018), PFC (three studies) (Junhai et al., 2008; Guoqiang et al., 2014; Makary et al., 2018), and ACC (three studies) (Ji et al., 2007; Junhai et al., 2008; Makary et al., 2018).

Correlation analyses between resting-state brain activity and behavior measurements showed that increased FC in default mode network (DMN) and PAG–amygdala were associated with decreased pain scores in LBP patients after acupuncture (Li et al., 2014; Yu et al., 2020). Moreover, there were significant correlations between the task-state fMRI signal in the left insula, STG, right supramarginal gyrus, SI, and VAS (Makary et al., 2018).

## Discussion

Nineteen articles that used MRI to investigate the central mechanism of acupuncture were enrolled in this review. In the past 14 years, using neuroimaging technologies to explore the central mechanism of acupuncture for treating LBP has attracted increasing attention. This review aims to analyze the methodology issues and study results by the systematic review of 19 neuroimaging papers on acupuncture for LBP so as to provide reference to deeply understand the current status and approaches for future studies.

### Functional and Structural Abnormalities in the Brain of LBP Patients

In this review, five studies reported that LBP patients showed cerebral functional and structural alterations compared with HC. The structural alterations mainly include the elevated GVM in the SI and reduced FA in SI–back regions. The functional changes usually manifest as reduced functional connectivities of DMN; higher ReHo values in the bilateral inferior temporal gyrus, left STG, and left superior parietal gyrus; and lower ReHo values in bilateral postcentral gyrus, bilateral superior frontal gyrus, and right supplementary motor area. Furthermore, a systematic review on neuroimaging studies of LBP patients indicated that brain regions such as the ACC, insula, mPFC, and cerebellum were involved in the central pathological changes of LBP patients (Kregel et al., 2015). These studies mapped the functional and structural alterations in LBP patients and provided the potential target for exploring the central responses to acupuncture stimulation in LBP patients.

### Acupuncture-Induced Brain Alterations in LBP Patients

**Figure 3:**
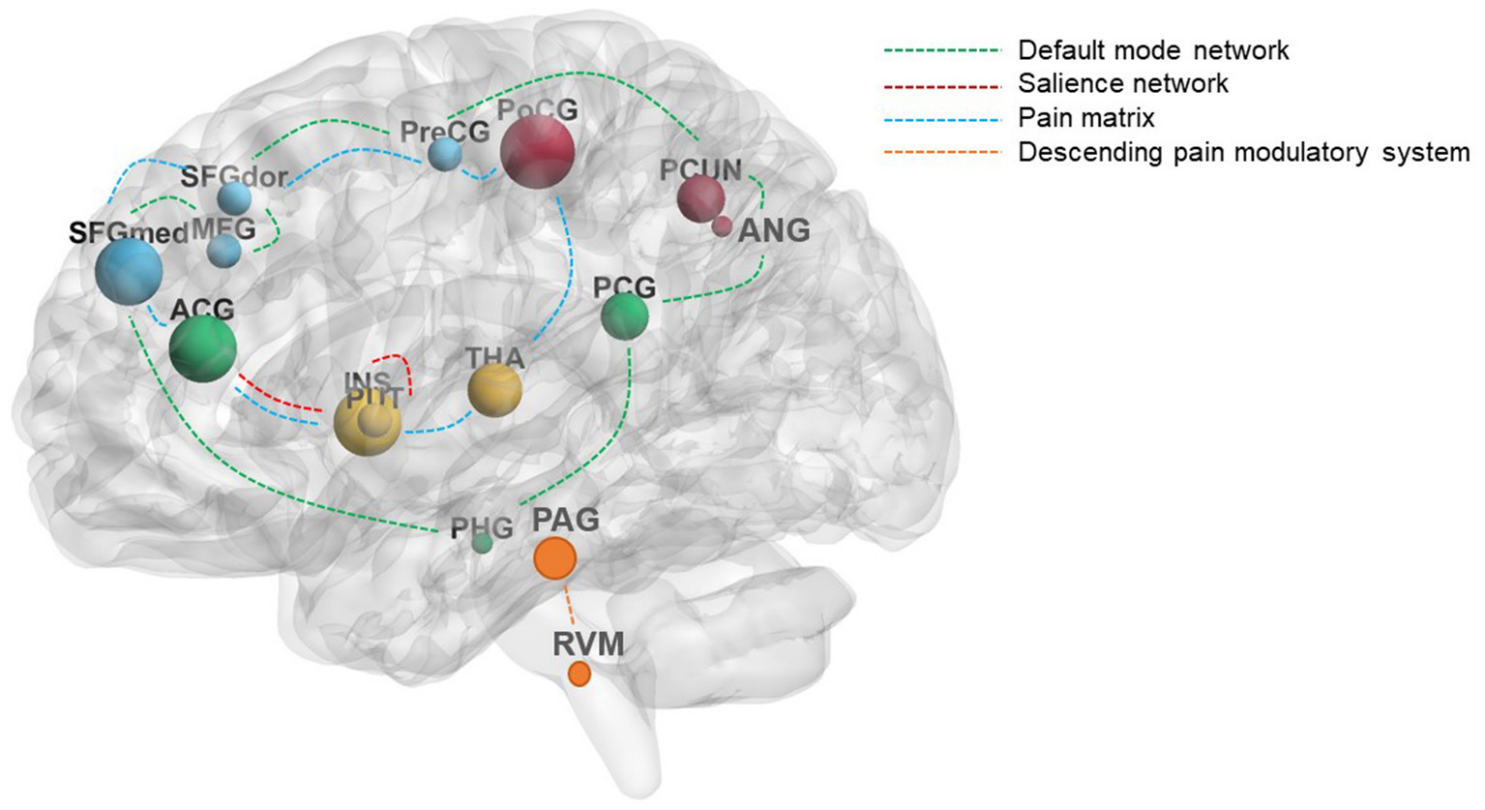
The main reported brain regions induced by acupuncture in LBP patients. The size of the nodes represents the frequency of the brain regions; the different colors of the dashed lines represent the different modulation pathways of acupuncture. The green dashed lines mainly represent the default mode network, the red dashed lines mainly represent the salience network, the blue dashed lines mainly represent the pain matrix, and the yellow dashed lines mainly represent the descending pain modulatory system. ACG, cingulate gyrus, anterior part; ANG, angular gyrus; INS, insula; LBP, low back pain; MFG, middle frontal gyrus; PAG, periaqueductal gray; PCG, cingulate gyrus, posterior part; PCUN, precuneus; PHG, parahippocampus; PoCG, postcentral gyrus; PreCG, precentral gyrus; PUT, putamen; RVM, rostral ventral medulla; SFGdor, superior frontal gyrus, dorsolateral; SFGmed, superior frontal gyrus, medial; THA, thalamus.

For LBP patients, acupuncture can elicit widely cerebral responses. These brain regions included the PFC (middle frontal gyrus and superior frontal gyrus), precentral gyrus, ACC, PCC, insula, thalamus, postcentral gyrus, putamen, precuneus, angular gyrus, parahippocampus, PAG, rostral ventral medulla (RVM), posterior inferior parietal lobe, and cerebellum regions and were mainly distributed in the “pain matrix,” DMN, salience network (SN), and descending pain modulatory system (DPMS) (Figure 3). Previous studies have reported the abnormal connectivity in DMN and SN in pain disorders (Zhao et al., 2017; Tu et al., 2020). In this review, several studies found that acupuncture can positively regulate the function of DMN and SN (Li et al., 2012, 2014; Lee et al., 2019; Xiang et al., 2019; Yan et al., 2019). Multiple neuroimaging studies suggested that modulating the activity of DMN is an important mechanism of acupuncture therapy (Deng et al., 2016; Fu et al., 2017; Sun et al., 2021) and regulating the cerebral function of the “pain matrix” is the common characteristic of acupuncture analgesia (Zhao et al., 2014; Shen et al., 2019). Thus, improving abnormal structural and functional activities in the brain of the LBP patient is an important mechanism of acupuncture treatment for LBP. In addition, abundant evidence suggests that modulating the DPMS, comprising the PAG and RVM, is one of the mechanisms of acupuncture analgesia (Chen et al., 2015; Li et al., 2016).

Among dozens of brain regions which responded to acupuncture for treating LBP, the PFC, insula, cerebellum, SI, and ACC were the main reported brain regions. The PFC and ACC are not only the main nodes of the “pain matrix” but also the key regions in DMN. They participate in pain modulatory, pain anticipation, affective, and cognitive processing (Tracey and Mantyh, 2007; Qin et al., 2008). It is interesting that Makary et al. reported that activation in ACC was unique to verum acupuncture and that activation in the PFC was observed in the sham acupuncture (Makary et al., 2018) and significantly correlated with the belief in acupuncture effectiveness score (Makary et al., 2018). Actually, the PFC is believed to be linked with expectancy-related modulation of pain processing (Casey, 1999). As a key region in the pain matrix and SN, the insula plays an important role in the cognitive and affective perception of pain (Kong et al., 2006), manifests a significant activation in chronic pain (Ihara et al., 2019) and experimental pain (Peyron and Fauchon, 2019), and is widely involved in acupuncture analgesia (Chae et al., 2013). In this review, nine studies identified the participation of insula in acupuncture for treating LBP. The cerebellum plays an important role in sensorimotor and vestibular control and also participates in cognition, autonomic, and emotional control (Schmahmann, 2019). Hui et al. reported that activation of the cerebellum elicited by pain occurred during the acupuncture stimulations (Hui et al., 2005). The SI cortex is a major component in the pain matrix participating in pain localization and discrimination. Kim et al. found that 4 weeks of acupuncture treatment for LBP can normalize the anatomic alterations of both gray matter (GM) and white matter (WM) in SI including decreasing the GVM and increasing the FA and AD. A similar structural modulation in SI was reported in a neuroimaging study on acupuncture for treating carpal tunnel syndrome (Maeda et al., 2017).

Generally, the majority of acupuncture-neuroimaging studies focus on the functional changes resulting from acupuncture stimulation, while a few studies centered on the structural alteration induced by acupuncture intervention. Among the 19 studies included in this review, only two studies reported the improvements in altered GM/WM of LBP patients. This phenomenon of emphasizing the function over structure in acupuncture-neuroimaging studies is closely related to the characteristic that acupuncture is good at regulating functional abnormalities. The few studies on acupuncture influencing the brain structure provide valuable evidence for the promotion of structural plasticity in the central nervous system (CNS) by acupuncture. Future studies could pay more attention to the structural plasticity in the CNS induced by acupuncture and take the accumulation effect of long-term acupuncture treatment and the cerebral structural changes in physiological period into consideration.

### The Brain Functional Alterations Induced by MA and EA

MA and EA are the two main modalities in acupuncture clinic practice. The advantages of MA are traditional and convenient, while the strong point of EA is quantifiable. Some studies hold that EA was more effective than MA in analgesia (Kong et al., 2002), while some studies hold that MA produced a better-sustained effect than EA (Schliessbach et al., 2011). In this review, among the 17 studies on acupuncture regulating the cerebral function in LBP patients, 13 studies selected MA as acupuncture intervention and four studies used EA. Either MA or EA could elicit the cerebral activity changes in the PFC, insula, SI, and ACC. These regions all belong to the “pain matrix” and the high-frequency brain regions in acupuncture-neuroimaging for pain. It is a pity that there was no published study which investigated the similarities and differences in cerebral responses between MA and EA in LBP patients. Some studies on healthy subjects have shown that both EA and MA could activate the PFC and insula (Kong et al., 2002; Jiang et al., 2013), EA produces more widespread fMRI signal changes than MA (Napadow et al., 2005), and EA can produce more activation and less deactivation than MA (Li et al., 2003; Schliessbach et al., 2011). These studies on healthy subjects suggest that different brain mechanisms may be recruited during MA and EA (Li et al., 2003).

### Acupuncture-Induced Brain Alterations in Resting-State and Task-State fMRI

Resting state and task state are the two main study designs in fMRI. The advantage of ts-fMRI is that it can directly reflect the effects of an explicit task in the brain, while the lack of tasks makes rs-fMRI quite simple in experimental design and easy to cooperate with patients. In this review, 13 studies used resting-state fMRI, and five studies used task-state fMRI. The common brain regions that were induced by acupuncture in resting-state and task-state fMRI were the PFC, insula, SI, SII, brainstem, and ACC. Generally, ts-fMRI is used to evaluate the instant effect of acupuncture analgesia, and rs-fMRI is applied to evaluate the cumulative effect of acupuncture. Notably, the angular gyrus was altered when evaluating the cumulative effect of acupuncture, but not when evaluating the instant effect. The angular gyrus is located in the anterolateral region of parietal lobe and is an important part of the DMN. Liu et al. (2021) found that there was a significantly greater ReHo value increase in the angular gyrus after the 12th acupuncture sessions compared with after the first acupuncture session in migraine patients, which indicated that the cumulative effect of acupuncture is more extensive and significant than the instant effect. This review showed that in the neuroimaging studies of acupuncture treatment for LBP, researchers not only pay attention to the instant effect of acupuncture but also pay attention to the cumulative effect of acupuncture.

In this review, five out of 13 rs-fMRI studies used ReHo as analytical approach. As an example of functional segregation, Reho has the advantages of simplicity, stability, and repeatability. However, since the brain is an integrated network rather than an isolated cluster, a functional integration approach is more popular nowadays. Therefore, future studies combining functional segregation with functional integration approach will help to better clarify the mechanism of acupuncture treatment for LBP.

## Conclusion

The neuroimaging studies associated with acupuncture treatment on LBP have been widely conducted. These studies covered the functional and structural changes elicited by acupuncture stimulation, included both EA and MA, and took the resting state and task state into consideration of the study design. The brain regions involved in acupuncture analgesia for LBP were mainly located in the pain matrix, DMN, SN, and DPMS, especially in the PFC, insula, cerebellum, SI, and ACC. However, acupuncture neuroimaging studies for LBP were only performed with MRI. In the future, the combination of multiple imaging technologies might be a new approach to deeply investigate the central mechanism of acupuncture for LBP.

## Data Availability Statement

The original contributions presented in the study are included in the article/supplementary material, further inquiries can be directed to the corresponding author.

## Author Contributions

FZ designed the study. XD and YQ performed the paper search, paper selection, and data extraction. QW, PM, RS, LL, TY, YL, and QX discussed the results and wrote the paper. All authors have read and approved the publication of the final manuscript.

## Funding

This work was supported by grants from the National Natural Science Foundation of China (Grant No. 81973960) and Sichuan Province Scientific and Technological Innovation Team for Youths (Grant No. 2019JDTD0011).

## Conflict of Interest

The authors declare that the research was conducted in the absence of any commercial or financial relationships that could be construed as a potential conflict of interest.

## Publisher's Note

All claims expressed in this article are solely those of the authors and do not necessarily represent those of their affiliated organizations, or those of the publisher, the editors and the reviewers. Any product that may be evaluated in this article, or claim that may be made by its manufacturer, is not guaranteed or endorsed by the publisher.
