# Supplementary material for: Neuroimaging Studies of Acupuncture on Low Back Pain: A Systematic Review
Source: Front Neurosci. 2021 Sep 20;15:730322. doi: 10.3389/fnins.2021.730322 (PMC8488100; doi:10.3389/fnins.2021.730322)
Supplement: Supplementary file 1 [file Data_Sheet_1.docx]

Supplementary Material

# Supplementary Figures and Tables

## Supplementary Tables

**Table S1.** Full search strategy for each of the electronic databases queried

| **Databases** | **Coverage** | **Searches** | **Hits** |
| --- | --- | --- | --- |
| PubMed Database | Date of inception - Dec 31th, 2020 | 1. (((((((neuroimaging[MeSH Terms]) OR (neuroimaging[All fields])) OR ((neuroimaging[MeSH Terms]) OR (neuroimaging[All fields]))) OR (("magnetic resonance imaging"[MeSH Terms]) AND ("magnetic resonance imaging"))) OR ((Positron-Emission Tomography[MeSH Terms]) AND (Positron-Emission Tomography))) OR ((functional magnetic resonance imaging) OR (fMRI) OR (functional image))) OR ((Diffusion Tensor Imaging) OR (DTI))) OR ((structural magnetic resonance imaging) OR (sMRI))  2. (Back Pain[Mesh] OR dorsalgia[Title/Abstract] OR backache[Title/Abstract] OR back pain[Title/Abstract] OR coccyx[Title/Abstract] OR coccydynia[Title/Abstract] OR sciatica[Title/Abstract] OR spondylosis[Title/Abstract] OR lumbago[Title/Abstract] OR back disorder*[Title/Abstract] OR sciatic neuropathy[Mesh] OR Back Muscles[Mesh] OR low back pain[Title/Abstract])  3. ((((((((((Acupuncture Therapy[MeSH Terms]) OR (acupuncture[MeSH Terms])) OR (Acupuncture Analgesia[MeSH Terms]))) OR (Acupuncture Therapy)) OR (Acupuncture)) OR (Acupuncture Points)) OR (acupressure)) OR (electroacupuncture)) OR (meridians)) OR (moxibustion)  4. #1 AND #2 AND #3 | 81 |
| EMBASE Database | Date of inception -Dec 31th, 2020 | #1. 'acupressure'/exp OR acupressure OR 'electroacupuncture'/exp OR electroacupuncture OR 'meridians'/exp OR meridians OR 'moxibustion'/exp OR moxibustion  #2. 'acupuncture therapy'/exp OR 'acupuncture therapy' OR 'acupuncture'/exp OR 'acupuncture' OR 'acupuncture points'/exp OR 'acupuncture points'  #3. acupoint$  #4. meridian$  #5. 'deqi'/exp OR deqi  #6. moxibustion$  #7. #1 OR #2 OR #3 OR #4 OR #5 OR #6  #8. 'backache'/exp  #9. dorsalgia OR ischialgia OR back pain OR lumbar adj pain OR sciatica OR coccyx spondylosis OR lumbago OR back disorder  #10. #7 AND #8 AND #9  #11. 'neuroimaging'/exp OR neuroimaging OR 'magnetic resonance imaging'/exp OR 'magnetic resonance imaging' OR 'mri'/exp OR mri OR 'structural magnetic resonance imaging'/exp OR 'structural magnetic resonance imaging' OR smri OR 'diffusion tensor imaging'/exp OR 'diffusion tensor imaging' OR 'dti' OR 'functional magnetic resonance imaging'/exp OR 'functional magnetic resonance imaging' OR 'fmri'/exp OR 'fmri' OR 'positron emission tomograph' OR 'pet'/exp OR pet  #12. #10 AND #11 | 31 |
| Cochrane database | Date of inception - Dec 31th, 2020 | #1 “Acupuncture Therapy” or “Acupuncture” or “Acupuncture Points”  #2 acupressure or electroacupuncture or meridians or moxibustion  #3 acupoint$  #4 meridian$  #5 deqi  #6 moxibustion$  #7 MeSH descriptor: [Back Pain] explode all trees  #8 dorsalgia  #9 backache or back pain  #10 (lumbar near pain) or (coccyx) or (coccydynia) or (sciatica) or (spondylosis)  #11 MeSH descriptor: [Sciatica] explode all trees  #12 (lumbago) or (discitis) or (disc near herniat*)  #13 neuroimaging OR ‘magnetic resonance imaging' OR mri OR 'structural magnetic resonance imaging' OR smri OR 'Diffusion Tensor Imaging' OR 'DTI' OR ‘functional magnetic resonance imaging’ OR ‘fmri’ OR 'positron emission tomograph' OR pet  #14 #1 OR #2 OR #3 OR #4 OR #5 OR #6  #15 #7 OR #8 OR #9 OR #10 OR #11 OR #12  #16 #14 AND #15  #17 #13 AND #16 | 51 |
| China National Knowledge Infrastructure (CNKI, Chinese Database) | Date of inception - Dec 31th, 2020 | (((((SU = 针灸) OR (SU = 针刺)) OR (SU = 电针)) OR (SU = 针法)) AND ((SU = 腰痛) OR (SU = 腰椎间盘突出) OR (SU = 坐骨神经痛) OR (SU = 腰腿痛)) AND (((((((SU = 神经影像) OR (SU = 脑影像)) OR (SU = 磁共振技术)) OR (SU = 功能磁共振)) OR (SU = 结构磁共振)) OR (SU = 弥散张量成像)) OR (SU = PET))) [Journal article] | 11 |
| Chinese Biomedical Literature Database  (CBM, Chinese Database) | Date of inception - Dec 31th, 2020 | (("针刺"[核心字段:智能] OR "针灸"[核心字段:智能] OR "电针"[核心字段:智能] OR "针法"[核心字段:智能]) AND ( "腰痛"[核心字段:智能] OR "腰椎间盘突出"[核心字段:智能] OR "坐骨神经痛"[核心字段:智能] OR "腰腿痛"[核心字段:智能])) AND ("神经影像"[核心字段:智能] OR "脑成像"[核心字段:智能] OR "磁共振技术"[核心字段:智能] OR "功能磁共振"[核心字段:智能] OR "结构磁共振"[核心字段:智能] OR "弥散张量成像"[核心字段:智能] OR "PET"[核心字段:智能]) | 2 |
| Chongqing VIP Database  (VIP, Chinese Database) | Date of inception - Dec 31th, 2020 | (K=腰痛 OR K=腰椎间盘突出 OR K=坐骨神经痛 OR K=腰腿痛) AND (((((K=神经影像 OR K=脑影像) OR K=磁共振技术) OR K=功能磁共振) OR K=结构磁共振) OR K=PET) AND (((K=针刺 OR K=针法) OR K=电针) OR K=针灸) | 4 |
| Wanfang Database  (WF, Chinese Database) | Date of inception - Dec 31th, 2020 | ((针刺 or 针灸 or 针法 or 电针) and (腰痛 or 腰椎间盘突出 or 坐骨神经痛 or 腰腿痛) and (神经影像 or 脑影像 or 磁共振技术 or 功能磁共振 or 结构磁共振 or 弥散张量成像 or PET)) | 130 |

Table S2. The results of risk of bias assessment of randomised controlled trials by the RoB 2

| **No.** | **Author (year)** | **Bias arising from the randomization process** | **Bias due to deviations from intended interventions** | **Bias due to missing outcome data** | **Bias in measurement of the outcome** | **Bias in selection of the reported result** | **Overall risk of bias** |
| --- | --- | --- | --- | --- | --- | --- | --- |
| 6 | Chen (2014) | Some concerns | Low | Some concerns | Low | Low | Some concerns |
| 8 | Guo (2015) | Some concerns | Low | Some concerns | Low | Low | Some concerns |
| 10 | Xiao (2017) | Some concerns | Low | Some concerns | Low | Low | Some concerns |
| 11 | Makary (2018) | Some concerns | Low | Low | Low | Low | Some concerns |
| 12 | Tu (2019) | Low | Low | Low | Low | Low | Low |
| 14 | Lee (2019) | Some concerns | Low | Low | Low | Low | Some concerns |
| 16 | Yu (2020) | Low | Low | Low | Low | Low | Low |
| 18 | Kim (2020) | Some concerns | Low | Low | Low | Low | Some concerns |
| 19 | Guan (2020) | Some concerns | Low | Low | Low | Low | Some concerns |

Table S3.The results of risk of bias assessment of non-randomised controlled trials by the ROBINS-I

| **No.** | **Author (year)** | **Bias due to confounding** | **Bias in selection of participants into study** | **Bias in classification of interventions** | **Bias due to deviations from intended interventions** | **Bias due to missing data** | **Bias in measurement of outcomes** | **Bias in selection of the reported result** | **Overall risk of bias** |
| --- | --- | --- | --- | --- | --- | --- | --- | --- | --- |
| 1 | Li (2007) | Low | Low | Low | Low | Low | Low | Low | Low |
| 2 | Zhang (2008) | Low | Low | Low | Low | Low | Low | Low | Low |
| 3 | Ye (2011) | Low | Low | Low | Low | Low | Low | Low | Low |
| 4 | Li (2012) | Low | Low | Low | Low | Low | Low | Low | Low |
| 5 | Li (2014) | Low | Low | Low | Low | Low | Low | Low | Low |
| 7 | Yu (2014) | Low | Low | Low | Low | No information | Low | Low | Low |
| 9 | Liu (2017) | Low | Low | Low | Low | Low | Low | Low | Low |
| 13 | Xiang (2019) | Low | Low | Low | Low | Low | Low | Low | Low |
| 15 | Zou (2019) | Low | Low | Low | Low | Low | Low | Low | Low |
| 17 | Liu (2020) | Low | Low | Low | Low | Low | Low | Low | Low |

## Supplementary Figures


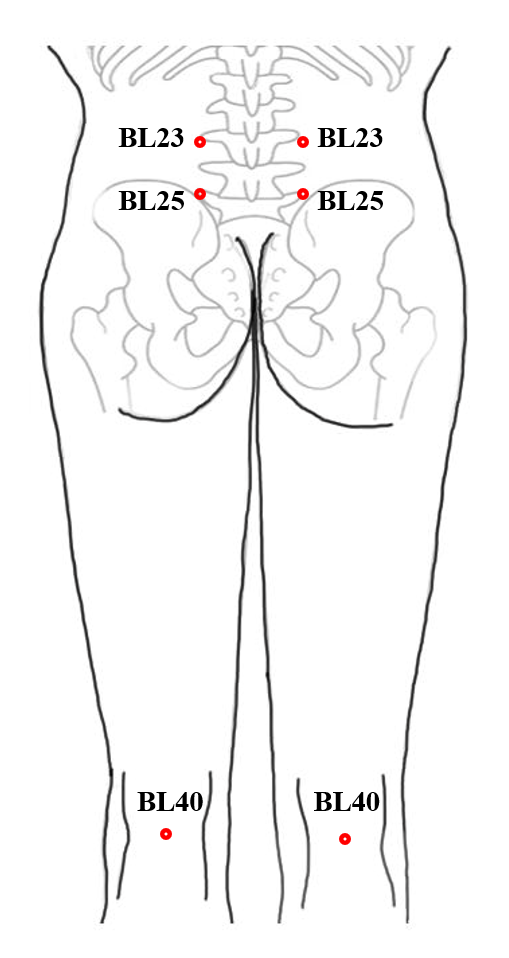


Figure S1. The most frequently used acupoints in acupuncture neuroimaging studies in LBP patients

BL23:1.5 cun lateral to the lower border of the 2nd lumbar vertebra spinous process; BL23:1.5 cun lateral to the lower border of the 4nd lumbar vertebra spinous process; BL40: Midpoint of the transverse crease of the popliteal crease.
